# Edge Server Selection with Round-Robin-Based Task Processing in Multiserver Mobile Edge Computing

**DOI:** 10.3390/s25113443

**Published:** 2025-05-30

**Authors:** Kahlan Aljobory, Mehmet Akif Yazici

**Affiliations:** 1 Information and Communications Research Group, Informatics Institute, Istanbul Technical University, 34469 Istanbul, Türkiye; yazicima@itu.edu.tr; 2 Department of Computer Science, Tikrit University, Tikrit 34001, Iraq; 3 Department of Computer Engineering, Istanbul Technical University, 34469 Istanbul, Türkiye

**Keywords:** edge computing, edge server selection, task offloading, computation delay, round robin, processor sharing

## Abstract

Mobile edge computing was conceived to address the increasing computing demand generated by users at the communication network edge. It is expected to play a significant role in next-generation (5G, 6G, and beyond) communication systems as new applications such as augmented/extended reality, teleoperations, telemedicine, and gaming become prolific. As the networks become denser, more and more edge servers are expected to be deployed, and the question of task offloading becomes more complicated. In this study, we present a framework for task offloading in the presence of multiple edge servers that employ round-robin task scheduling. Most studies in the literature attempt to optimize the offloading process under the assumption that each user generates just a single task, or they generate one task every time slot in a discrete-time system where all the tasks are handled within a slot. Furthermore, first-come-first-served queueing models are typically used in studies where queueing is considered at all. The work presented is novel in that we assume continuous and stochastic task arrivals generated by multiple users and round-robin task scheduling at the edge servers. This setting is considerably more realistic with respect to the existing works, and we demonstrate through extensive simulations that round-robin task scheduling significantly reduces task delay. We also present a comparison of a number of server selection mechanisms.

## 1. Introduction

Recently, there has been a significant increase in the use of resource-intensive applications such as face recognition, image/video processing, interactive gaming, and augmented reality on mobile devices. However, running such applications on mobile devices poses significant challenges due to their high resource demands. Offloading these tasks to the cloud, as suggested by [1], offers some relief, but it is not suitable for latency-sensitive applications. To address these challenges, mobile edge computing (MEC) has emerged [2], allowing computationally intensive applications to be offloaded to edge servers (ESs) located at the edge of the mobile network, such as base stations (BSs). This approach effectively supports both computationally intensive and latency-sensitive applications [3].

A significant challenge faced by MEC systems is the need to manage highly dynamic workloads with their relatively limited computational power. Queueing models provide a systematic approach to address this issue by facilitating the performance evaluation of resource allocation strategies. One crucial application of queueing models in MEC is the analysis of latency. In time-sensitive applications such as augmented reality, any processing delay can negatively impact the user experience. Queueing models offer insights into expected waiting and processing times, enabling system designers to minimize delays by optimizing server configurations or task scheduling policies. For example, by implementing a priority-based queueing model, as suggested in [4,5], high-priority tasks like health-monitoring devices can be processed ahead of less critical tasks, ensuring adherence to stringent latency requirements.

In the presence of multiple ESs, selecting the right server for offloaded computations poses a distinct challenge compared to cloud systems. Unlike the expansive server farms in the cloud, ESs have relatively limited resources, making server selection a critical task. In MEC, this complexity is compounded by the limited coverage of ESs. Users must choose between going to the nearest server, selecting a server with a lighter load, or, in certain cases, migrating tasks to a new server after they are already offloaded, which might save computation costs but introduces switching overhead. This decision is influenced by factors such as server workload, backhaul communication delay, and the available computational capacity. Therefore, an optimal server selection strategy must minimize the overall costs, including communication, computation, and switching expenses.

Addressing the complexities of extending server selection to multiple users reveals intricate challenges. Offloading computation from one user increases the workload on the designated server, thereby affecting the computational efficiency of all the users dependent on that server. The need for uninterrupted user service and the dynamic nature of user mobility further complicate server selection. User movement alters the communication costs across different regions or BSs, and the continuous arrival and departure of tasks shift ES workloads. Relying solely on the instantaneous status of the system for decision-making often yields inefficient results. Instead, an optimal server selection strategy must focus on the long term, requiring the estimation of various costs within this dynamic environment.

The existing literature predominantly explores two key server selection methods: the nearest server approach and the optimal server approach (based on a specific criterion). However, a comprehensive comparison to determine their relative superiority remains absent. Our previous work [6] provided an initial comparison of three distinct server selection methods to begin addressing this gap. Building on that foundation, this paper expands the scope of our prior study to address the aforementioned challenges comprehensively. We conduct a comparative analysis of the three approaches introduced in our earlier work, alongside a random selection scheme, and evaluate them against the method proposed in [7] as a benchmark. Our model considers mobile users generating computationally intensive tasks within a network comprising multiple BSs. Given the dense deployment of small cells in 5G networks, equipping all the cells with wired backhaul is neither feasible nor cost-effective due to the challenges associated with fiber optic backhaul installation [8]. As highlighted in [9], wireless backhaul presents a more practical and economical alternative. Leveraging advancements in millimeter-wave (mmWave) technologies, wireless backhaul emerges as a viable substitute for wired solutions. Consequently, integrating wireless backhaul into our system becomes a priority. We examine a system in which a central controller selects the server to handle each generated task based on factors such as task size, required CPU cycles, and the CPU capacities of the ES. This controller plays a critical role in the decision-making process by also accounting for communication delays and computation delays. The main contributions of this paper are as follows:

- Most studies in the literature assume one task per user and optimize the offloading process based on this. We, on the other hand, assume continuous and stochastic arrival of tasks, which is much more realistic.
- Again, most studies in the literature either allocate fixed and dedicated CPU cycles (frequencies) to each task or rely on the first-come-first-served (FCFS) queueing model. Our approach adopts round-robin process scheduling, which is both more realistic and resource-efficient. To the best of our knowledge, this is the first study considering round-robin scheduling in the context of MEC.
- We perform a comparative analysis of four distinct ES selection methods, evaluating their performance in terms of average task sojourn time. Among these methods, “nearest server” and “random selection” are straight-forward heuristics and serve as benchmarks. The other two, “least remaining CPU cycles” and “fewest active tasks”, can be interpreted as variants of the “join the shortest queue” policy, which typically provides load balancing in multiserver queueing systems.
- The numerical results for performance comparison and assessment are based on a real-world dataset of the city of Oulu, Finland.

In summary, we describe an offloading decision scheme in a multiserver MEC system in which the servers employ round-robin scheduling. The two main technical novelties of our work include the investigation of a multiserver MEC system under round-robin scheduling and the comparison of server selection schemes in this scenario. Although the server selection schemes themselves are not new, their comparison in this specific setting is novel.

The structure of the remainder of this paper is as follows: Section 2 provides an overview of the related works. Section 3 describes the system model in detail. The performance evaluation of the proposed approach, including comparisons with benchmark schemes, is presented in Section 4, and, finally, we draw our conclusions in Section 5.

## 2. Related Works

Numerous user offloading decision methods have been developed to optimize server selection for task offloading, with predetermined server locations, focusing on minimizing the time and energy consumption of user devices and computing nodes. In [10], the authors addressed a task offloading scheduling problem using a Markov chain model. They introduced a stochastic scheduling decision rule for each state to minimize the average delay of tasks, although their study was limited to a single-user scenario. Building on this work, ref. [11] extended the framework to a multi-user environment and proposed a game-theoretic approach to task offloading. They formulated the problem as a multi-user task offloading game and derived the Nash equilibrium of the game. Meanwhile, the study in [12] emphasized energy efficiency in the design of computation offloading mechanisms, accounting for both wireless transmission energy and the computational energy required to offload user tasks to edge computing nodes. However, the methods proposed in [11,12] are not well suited to dense networks with a large number of BSs and users as they were designed for a single BS with multiple users. The minimization of the overall computation delay by optimizing the user task offloading and transmission time in a scenario involving multiple users and ESs employing non-orthogonal multiple access (NOMA) for communications is explored in [13].

Regarding server placement and selection, numerous studies in the literature have proposed diverse methodologies, ranging from machine learning algorithms and optimization models to dynamically choose servers based on network conditions, workload characteristics, and Quality of Service (QoS) requirements. The study in [14] proposed the GASS (Genetic Algorithm and Simulated Annealing Algorithm for edge server selection), an integrated approach designed to optimize server selection while minimizing time and energy overheads. Similarly, ref. [15] introduced a novel server selection algorithm for edge computing, aiming to identify the most suitable MEC server for initiating services during the service request process. This algorithm considers factors such as resource availability, MEC server capabilities, and communication channel conditions between users and MEC servers. In [16], the problem of continuous server selection in a time-slotted system with every user producing a task every slot was formulated as a Markov decision process (MDP). However, the reliance on future knowledge, which is often unattainable through traditional methods, poses a significant challenge. To overcome this, the study leverages deep reinforcement learning (DRL) with a long short-term memory (LSTM)-based neural network, enabling the model to learn from past server selections and infer critical dynamic factors for decision-making. To address how to use DRL to deploy application engines in an efficient manner on distributed fog servers, the work in [17] proposed XDDRL (experience-sharing distributed deep reinforcement learning) to well address how the DRL can consider complex interactions of directed acyclic graph-structured IoT applications and fog server resources. For the challenge of adopting an optimal albeit computationally expensive solution, ref. [18] proposed a DRL-based IoT application scheduling algorithm. This approach dynamically improves the response time of heterogeneous IoT applications but also scales the load across ESs effectively. In [19], the authors proposed a comprehensive approach that integrates ES selection and service placement, aiming to maximize the overall profit across ESs while considering constraints such as the number of servers, inter-server relationships, storage, and computing capacity. Their solution employs a two-step method involving clustering algorithms and nonlinear programming. Similarly, ref. [20] introduced a service deployment strategy focused on load and service popularity, intending to minimize response delays for service requests. This strategy prioritizes service popularity and server load, formulating a deployment model that is solved using an enhanced ant colony algorithm to achieve optimal service placement. Meanwhile, ref. [21] presented a measurement-based server selection method designed to identify the server with the shortest delay. The performance of this method, evaluated in terms of selection accuracy and delay, is assessed through simulations. The authors assume that the storage server is selected based on the fastest response time observed over a number of measurements, where the measurements are derived from the request/response round trip time. Other studies [22,23,24,25] proposed optimization problems to minimize the energy consumption of UE and/or ESs while considering the resource allocations and delay requirements of the sensitive offloaded tasks in an edge–cloud-assisted environment. Previous studies [26,27,28,29] focused on edge computing systems without remote cloud support. Specifically, Ref. [26] introduced a framework for jointly optimizing radio and computational resources, balancing energy consumption and latency. Ref. [27] addressed the joint optimization of offloading decisions and bandwidth allocation to achieve near-optimal offloading performance. Ref. [28] aimed to maximize the weighted sum computation rate across all UE by jointly optimizing resource allocation and energy consumption. Similarly, ref. [29] explored the co-optimization of computing mode selection for individual UE and transmission time allocation within the system. Numerous other studies have proposed diverse approaches to address key challenges in MEC related to energy efficiency [30,31], offloading decisions [32], tasks allocation and execution latency [33], and communication capacity optimization [34].

The common themes throughout the studies in the literature can be summarized as follows:

- Some studies assume that users produce a single shot of a task, and an optimization problem is formulated based on this snapshot-like system frozen in time. Many of these optimization problems are intractable and an approximate solution via machine learning tools is proposed.
- Some studies attempt to extend this approach to dynamic time-dependent scenarios. Most of these assume discrete-time-slotted systems, where every time slot is treated as an optimization problem. Typically, these studies rely on Markov decision processes to obtain results.
- Most studies involving optimization problems consider the solution in terms of assigning CPU frequency slices to each task. Although suitable for the solutions of snapshot systems, this approach would lead to inefficiencies if tasks are allowed to be generated over time, particularly in a stochastic manner as the arrival process is typically ignored in the formulations.
- A limited number of studies consider stochastic task arrivals. Elementary queueing models are usually employed in these studies to model task delays. However, FCFS scheduling is typically assumed in such studies, which is not the typical application in real-life offloading scenarios.

There are also a number of studies that investigate systems in which an MEC system is coupled with either back-up cloud servers or physical-layer operations such as beamforming or NOMA. We focus our attention purely on the dynamics of the ES selection, without any cloud servers or physical-layer components. From this point of view, Table 1 summarizes the works investigated with regard to the key aspects of our model. The system we analyze is unique in involving multiple ESs employing round-robin scheduling and multiple mobile users generating tasks in continuous time in a stochastic manner.

**Table 1:** Comparison of related works in terms of key aspects.

Publication	Tasks	Users	Edge Servers	Cloud Server	Mobility
[11]	Single	Multiple	Single	No	No
[22]	Single	Multiple	Single	Yes	No
[23]	Single	Multiple	Multiple	Yes	No
[24]	Multiple	Multiple	Single	Yes	No
[25]	Multiple	Multiple	Multiple	Yes	No
[26]	Single	Single	Single	No	No
[27]	Multiple	Multiple	Single	No	No
[28]	Single	Multiple	Single	No	No
[29]	Single	Multiple	Single	No	No
[30]	Single	Single	Multiple	No	No
[31]	Single	Single	Single	No	No
[32]	Single	Multiple	Single	No	No
[33]	Multiple	Single	Multiple	No	No
[34]	Single	Multiple	Multiple	No	No
[7]	Multiple	Multiple	Multiple	No	No
Our Work	Multiple	Multiple	Multiple	No	Yes

## 3. System Model

We consider a mobile edge computing system comprising a set of M BSs, denoted as $B=\{B_{1},B_{2},\ldots ,B_{M}\}$. These BSs are interconnected via a high-speed wireless backhaul link, enabling fast communication and efficient data exchange [35]. Deploying a server at every BS is not only impractical due to the substantial deployment costs for the service provider (SP) but also resource wasting. In a practical network scenario, users typically are not distributed uniformly across the coverage area, resulting in imbalanced resource demands. Certain locations, such as public libraries, shopping malls, and tourist attraction points, naturally attract higher user densities compared to others. Consequently, strategically allocating computing resources to these high-demand areas can enhance overall network performance and reduce operational costs for the SP. This rationale underpins our decision not to deploy ESs at every BS within the network. Our system operates with a set of V servers, represented as $S=\{S_{1},S_{2},S_{3},\ldots ,S_{V}\}$.

Our system involves multiple (*N*) users who are not stationary and navigate the network, running diverse applications such as virtual reality, augmented reality, image/video processing, and online gaming. These applications generate tasks that may be offloaded to the servers to minimize sojourn time. In this setup, a centralized server with robust computational capabilities acts as the network controller. When a mobile device generates a task, it sends a request to the controller containing task details, such as size, required CPU cycles, and the CPU capacity of the mobile. The controller processes this information, estimates the task completion delay across the candidate ESs, makes the offloading decision in terms of whether to offload or not, and, in case of offloading, selects the appropriate server. The estimated delay accounts for factors such as communication channel delay, task migration delay over the wireless backhaul, computation delay at the server, and queueing delay. The estimated delay is then compared to the local computation delay on the mobile device. Queueing delay at the mobile device is disregarded as task arrivals per user are assumed to be light. The offloading decision made by the controller is then communicated back to the mobile device. The delay incurred by this decision process is also disregarded, which is reasonable because

- the controller can monitor the states of the ESs (in terms of how loaded they are) through periodic updates using very small messages;
- the task characteristics can be communicated to the controller using very small messages, mostly over the backhaul;
- as will be explained in the upcoming subsections, the offloading decision is based on simple calculations and not heavy computation like any machine learning tools.

If a task is to be offloaded, and the selected ES is not hosted at the BS serving the mobile device that the task was generated at, the task is forwarded from the serving BS to the BS where the chosen ES is deployed through the wireless backhaul link. If a task is offloaded and the associated user switches to a new BS due to mobility, the output of the completed task is sent through the backhaul to the new serving BS, which then communicates it to the user. Unlike most studies in the literature that overlook the delay involved in transmitting the results back to the user, this paper considers both the time required to transmit the results over the backhaul link (when the ES is not deployed at the serving BS) and the transmission delay over the communication channel between the serving BS and the mobile user.

**Figure 1:**
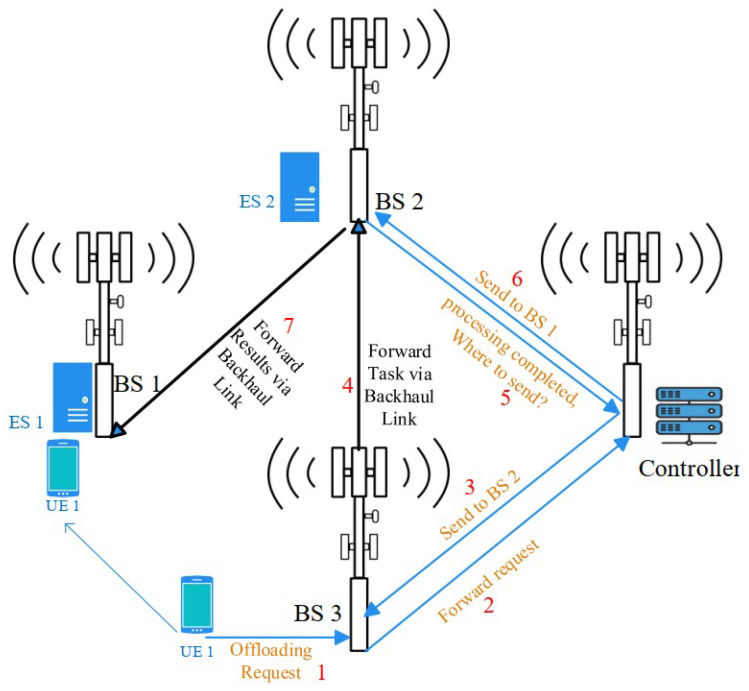
Multiserver mobile edge computing sample scenario.

A representative scenario illustrating this procedure is shown in Figure 1. User equipment (UE) 1 initially connects to BS 3 and generates a task. UE 1 queries the controller with relevant information for an offloading decision. In response, the controller assigns the task to ES 2, hosted at BS 2. Consequently, the task data are first sent to BS 3 via the communication channel and then forwarded to ES 2 via the wireless backhaul. During the task execution, UE 1 moves and is handed over to BS 1 from BS 3. Once ES 2 completes the task, it queries the controller for UE 1’s current location. The controller informs ES 2 that UE 1 is now connected to BS 1. Finally, the task result is communicated over the backhaul to BS 1, and from there to UE 1 via the communication channel.

In the remainder of this section, we detail the task characteristics, user mobility, communication, computation, and queueing models, and describe the offloading decision and server selection methods. In Section 4, where we provide the numerical performance results, we use [7] as a benchmark for performance comparison. We lay out the main differences in the system model of this benchmark from ours in Section 3.6.

### 3.1. Task Characteristic Model

A task is defined by the volume of the data it needs to process (denoted *D*), measured in bits, and the number of CPU cycles required to complete the processing. This is typically represented as the number of CPU cycles needed per bit of data (denoted *C*), which varies depending on the specific task [36]. Each mobile device is assumed to generate tasks according to independent and identically distributed Poisson processes with a rate parameter of λ. Therefore, the aggregate task arrival process into the entire system is also Poisson with an arrival rate of λN.

### 3.2. Mobility Model

We assume that users are moving according to the random waypoint (RWP) mobility model. Users are initially placed at random locations across the simulation area. They begin their trajectories by selecting destinations uniformly at random across the simulation area. Each user moves towards its destination at a random speed uniformly chosen between 0 and 50 m per second. Once the user reaches the destination, it pauses at that location for a random duration chosen uniformly between 0 and 10 s. After this pause, the user randomly selects a new destination and repeats this process for the duration of the simulation. The random selections for the destinations, speeds, and pause times are all independent.

### 3.3. Communication Model

When two parties (whether a UE and a BS or two BSs) are communicating, the received signal power (in dBm) at the destination, denoted as $P_{R}$, is given by

(1) $$P_{R}=P_{T}+G-L+\bar{X},$$

where $P_{T}$ represents the transmitted signal power in dBm, *G* is the receiver antenna gain in dB, *L* is the path loss in dB, and $\bar{X}$ accounts for the zero-mean log-normally distributed shadow fading effect, with a standard deviation of $\sigma_{SF}$.

For the path loss *L*, we utilize the Urban Macrocell (UMa) non-line-of-sight (optional) case from the 3GPP technical report [37]. The path loss in dB is given by

(2) $$L=32.4+20\;\log_{10}\left(f_{c}\right)+30\;\log_{10}\left(d_{3D}\right),$$

where $f_{c}$ represents the carrier (central) frequency, and $d_{3D}$ denotes the three-dimensional distance between the communicating entities. This path loss expression highlights the importance of accounting for the actual transmitter and receiver antenna heights. The antenna heights of the BSs and UE are denoted by $h_{\mathrm{BS}}$ and $h_{\mathrm{UT}}$, respectively [37]. The signal-to-noise ratio (SNR) in dB, denoted as γ, is then given by

(3) $$\gamma =P_{R}-P_{N},$$

where $P_{N}$ represents the noise power in dBm.

Upon deciding to offload, the controller instructs the user to transfer the task data to the selected server, which will happen at a data rate of

(4) $$R=W\;\log_{2}\left(1+10^{\gamma /10}\right),$$

where *W* represents the bandwidth in Hz. Expression (4) is also used to compute the data rate between BSs in case of task migration, as well as the transmission of the result of the task back to the UE. Therefore, the delay due to the transmission of the data or the result of a task of size *D* bits is

(5) $$T_{t}=D/R.$$

### 3.4. Computation and Queueing Models

Tasks can either be executed locally on the mobile device or fully offloaded to the server based on the controller’s decision. The CPUs of mobile devices are assumed to have a single core operating at a frequency of $f_{m}$. It is further assumed that task arrival rates are low enough to avoid queueing delays when tasks are executed locally. Under this assumption, the computation delay for a task with a data size of *D* bits and requiring *C* CPU cycles per bit is given by

(6) $$T_{c}^{m}=\frac{D\;C}{f_{m}}.$$

On the other hand, the server is equipped with a CPU operating at a frequency of $f_{s}$, featuring $n_{c}$ cores, and benefits from a speed-up factor *p* due to advanced processor design, pipelining, and improved cache and memory performance [38]. This configuration provides an effective speed-up of $\left(f_{s}\;n_{c}\;p\right)/f_{m}$ for tasks processed on the ES compared to local execution on the mobile device (disregarding queueing delay).

Most studies in the literature assume that fixed proportions of the available CPU frequency are allocated to each task offloaded to an ES. While this assumption simplifies the construction of the optimization problems aimed at enhancing the performance of MEC systems under various scenarios, it inherently leads to inefficient resource utilization unless the ES operates at full capacity (i.e., serving enough offloaded tasks to utilize its frequency fully). Under such conditions, any unused CPU capacity is effectively wasted. In contrast, our study adopts a round-robin scheduling approach for tasks offloaded to ESs, ensuring that the full computational capacity of the ES is utilized at all times. This approach is also more realistic as round-robin or weighted round-robin scheduling—or their variations—is typically employed in task schedulers in actual operating systems [39]. However, this approach introduces stochasticity to the task execution times as they become dependent on future task arrivals, making them unpredictable in advance.

A well-suited mathematical framework for modeling round-robin scheduling is the processor sharing (PS) discipline [40]. In this model, all tasks in the queue receive service simultaneously, with equal rates that are inversely proportional to the number of tasks in the system. When a new task arrives, the service rate allocated to each task decreases; conversely, when a task departs, the service rate for the remaining tasks increases. The mean service time of a task in a PS system is known to be linearly proportional to its size [41] (p. 215).

In our model, there are multiple ESs that are assumed to employ round-robin schedulers. However, the task arrivals into the ESs are not stationary, depending on the server selection methods, to be described later. Therefore, it is very challenging to analytically model the time-varying dependence of the expected service time. For this purpose, we introduce a parameter *q* to represent the queueing delay coefficient for offloaded tasks, consistent with our earlier work [4,5].

A task with *D* bits of data to process, requiring *C* cycles per bit, can be executed on an otherwise idle ES in a duration of $\left(D\;C\right)/\left(p\;n_{c}\;f_{s}\right)$ s. Under round-robin scheduling for offloaded tasks, the expected computation delay for the same task is given by

(7) $$T_{c}^{s}=q\;\frac{D\;C}{p\;n_{c}\;f_{s}},$$

where q≥1. The value of *q* depends on the instantaneous load on the ES, which is influenced by factors such as the arrival rate, offloading decisions for each task, task size distribution, the number of CPU cycles required per bit for each task, and the CPU speed. As the system works, these change over time, and thus the *q* coefficients kept at each ES must be adaptively updated. The controller begins with initial *q* values for each ES and updates them after each task completion at the associated ES using damped averaging, as in

(8) $$q_{k+1}=\left(1-\theta \right)\;q_{k}+\theta \;\delta \;\frac{p\;n_{c}\;f_{s}}{D\;C},$$

where δ denotes the actual time a task spends at the ES, θ∈(0,1) is the damping coefficient, and *k* the time index of the completed task. Here, the quantity $\delta \;\frac{p\;n_{c}\;f_{s}}{D\;C}$ is the ratio of the actual time of a task spent at the ES to the time it would have spent if the ES were serving only this particular task (i.e., the task uses all available computing power and does not see any queueing delay). Therefore, this quantity represents the instantaneous *q* coefficient experienced by the latest completed task. In (8), the *q* value at the ES with which the task was executed is updated by the latest *q* value experienced.

Initially, it is assumed that the ES starts with an empty queue, ensuring no queueing delays for the first arriving tasks. Consequently, initializing $q_{0}=1$ for each ES is a reasonable starting point. After the departure of each task from an ES, its *q* value is updated according to (8). In case of a new task generation, the controller uses the current *q* value to estimate the expected service time of the task at each ES.

Considering local computation, the load on the mobile device is assumed to be sufficiently lower to overlook the queueing delay experienced by the tasks executed locally. Thus, we will concentrate on the queueing behavior and associated delays of the offloaded tasks under the PS discipline at the ESs.

### 3.5. Offloading and Edge Server Selection Models

When a task is generated, the controller makes the offloading decision with the goal of minimizing the task’s expected sojourn time. Based on the ES selection scheme (details to follow), a suitable ES candidate is identified. The sojourn times for local computation and offloading are then compared using the identified candidate ES. For a task with *D* bits of data and requiring *C* cycles per bit, the sojourn time in the case of local computation consists only of the computation delay, as given by (6). Conversely, if the task is offloaded, the sojourn time consists of the following:

- $T_{t}^{\left(1\right)}$: the transmission delay for sending the task data from the UE to its serving BS.
- $T_{t}^{\left(2\right)}$: if the selected ES is not hosted on the serving BS, the transmission delay for migrating the task data from the serving BS to the BS hosting the ES.
- $T_{c}^{s}$: the computation delay (including the queueing delay).
- $T_{t}^{\left(3\right)}$: if the selected ES is not hosted on the serving BS (at the time of the completion of the task, which may be different than the initial serving BS), the transmission delay for sending the task result from the ES to the serving BS.
- $T_{t}^{\left(4\right)}$: the transmission delay for sending the task result from the serving BS to the UE.

Thus, denoting the estimated sojourn time in case of task offloading with $T^{o}$, we have

(9) $$T^{o}=T_{t}^{\left(1\right)}+T_{t}^{\left(2\right)}+T_{c}^{s}+T_{t}^{\left(3\right)}+T_{t}^{\left(4\right)}.$$

As an example scenario, recalling Figure 1, the transmission delays involved would correspond to

$$\begin{array}{cccc} T_{t}^{\left(1\right)} & =\frac{D^{\left(1\right)}}{R^{\left(1\right)}},\; & T_{t}^{\left(2\right)} & =\frac{D^{\left(2\right)}}{R^{\left(2\right)}},\\ T_{t}^{\left(3\right)} & =\frac{D^{\left(3\right)}}{R^{\left(3\right)}},\; & T_{t}^{\left(4\right)} & =\frac{D^{\left(4\right)}}{R^{\left(4\right)}}, \end{array}$$

where $D^{\left(1\right)}$ and $D^{\left(2\right)}$ are equal to the task data size, $D^{\left(3\right)}$ and $D^{\left(4\right)}$ are the task result size, $R^{\left(1\right)}$ is the data rate between UE 1 and BS 3, $R^{\left(2\right)}$ is the data rate between BS 3 and BS 2, $R^{\left(3\right)}$ is the data rate between BS 2 and BS 1, and $R^{\left(4\right)}$ is the data rate between BS 1 and UE 1. Note that, if the selected ES is hosted at the serving BS at the time of task generation, $T_{t}^{\left(2\right)}$ would be zero, and, if the selected ES is hosted at the serving BS at the time of task completion, $T_{t}^{\left(3\right)}$ would be zero.

It should be noted that, unlike our previous work [6] and many others in the literature [42,43,44], the transmission delay at the backhaul as well as the delay incurred by transmitting the results of the execution back to the user is not neglected.

An important consideration for computing the data rate is that the controller is aware of the bandwidth *W*, the transmit power $P_{T}$, the antenna gain *G*, and the path loss *L* (based on knowledge of locations and antenna heights), but it cannot be assumed to know beforehand the amount of shadow fading a transmission will experience. Therefore, the controller assumes $\bar{X}=0$ in (1) and makes the offloading decision based on this imperfect knowledge. During the simulation study, however, a random value for the shadow fading loss is generated for each transmission after the offloading decision has been made. Thus, the obtained numerical results are more realistic.

In light of all these aspects, the offloading decision can be stated as

(10) $$\left\{\begin{array}{cc} \mathrm{execute}\;\mathrm{locally}, & \mathrm{if}\;T_{c}^{m}\leq T^{o},\\ \mathrm{offload}\;\mathrm{to}\;\mathrm{the}\;\mathrm{ES}\;\mathrm{selected}\;\mathrm{by}\;\mathrm{the}\;\mathrm{ES}\;\mathrm{selection}\;\mathrm{scheme}, & \mathrm{otherwise}, \end{array}\right.$$

where the definitions of $T_{c}^{m}$ and $T^{o}$ are given in (6) and (9), respectively. In Algorithm 1, we present the “Offloading Decision and Edge Server Selection Algorithm” (ODESSA). The helper method to compute the data rate between a given transmitter and receiver pair is presented in Algorithm 2, whereas the ES selection scheme is given in Algorithm 3.

**Algorithm 1** Offloading decision and edge server selection algorithm.

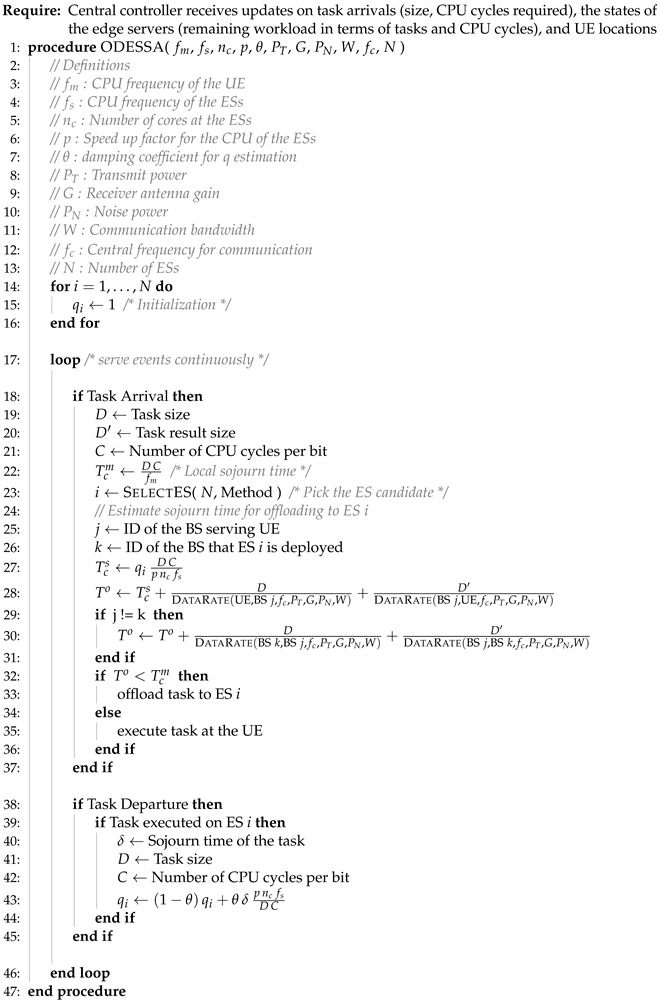



**Algorithm 2** The data rate between the transmitter Tx and the receiver Rx.

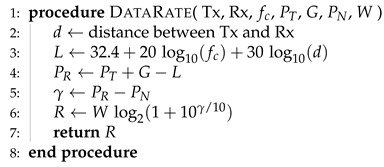



**Algorithm 3** Edge server selection scheme.

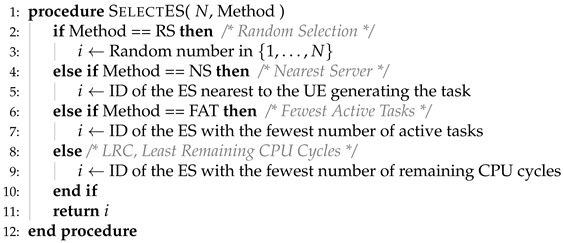



Next, we outline the four distinct ES selection schemes investigated in this study.

#### 3.5.1. Nearest Server (NS)

In this method, the controller calculates the Euclidean distance between the UE and each ES, designating the nearest ES to the user as the candidate server. In case multiple servers are deployed at the same BS and multiple such ESs turn out to be the nearest candidates, one of the idle candidates is chosen, if any. In such a case, we select the idle candidate with the smallest ES index number in the simulation study. This tie-breaking could also be achieved by a random selection among the idle candidates. If none of the closest servers is idle, the controller opts for the server with the fewest active tasks among the nearest servers.

#### 3.5.2. Least Remaining CPU Cycles (LRC)

In this method, the controller selects the ES that has the least amount of CPU cycles to execute. If multiple ESs are tied (typically when there are multiple idle ESs), the nearest candidate is selected. In case there are still ties, the candidate with the smallest ES index number or a random candidate can be selected.

#### 3.5.3. Fewest Active Tasks (FAT)

In this method, the controller selects the ES that has the fewest number of uncompleted tasks to be executed. If multiple ESs are tied (typically when there are multiple idle ESs), the nearest candidate is selected. In case there are still ties, the candidate with the smallest ES index number or a random candidate can be selected. Along with LRC, this method can be considered two variants of the well-known join the shortest queue (JSQ) policy [45].

#### 3.5.4. Random Selection (RS)

In this method, the controller (uniformly) randomly selects one of the ESs as the candidate, with no regard to the location of the UE, or the ES, or how loaded the ES is. This method is clearly very simple to implement and can be treated also as a benchmark for the aforementioned methods.

#### 3.5.5. Complexity of the ES Selection Schemes

Algorithm 3 presents the overall ES selection scheme. In general, efficient implementation of each of the four methods depends on the selection of appropriate data structures. The following remarks can be made regarding the complexities of server selection methods:

- NS requires the controller to find the nearest server to a UE at the time of an arrival. This can be achieved in Θ(logN) time, where *N* is the number of ESs, by building a *k*-d tree, which is a space-partitioning data structure.
- RS requires the selection of a server at random, and, hence, its complexity can be considered Θ(1).
- LRC and FAT require the controller to keep track of the workload on each of the servers in terms of either CPU cycles or tasks. The controller can store this information in a suitable data structure, such as a min-heap, so that finding the least-loaded server takes Θ(1) time. Updating the loads as time progresses is also straight-forward as all servers consume equal amounts of workload within equal durations. Upon an arrival, after the offloading decision, the workload of one server might be updated (i.e., increased by the offloaded workload). Again, using a suitable data structure such as a Fibonacci heap with an “increase-key” operation taking Θ(1) time, this operation is also quite efficient.

### 3.6. System Model of the Benchmark

The research in MEC is quite diverse, and several different scenarios have been examined in the literature, as overviewed in Section 2. In order to gauge the performance of the server selection methods in the context of the system model described in this section, we looked for candidate studies with similar/compatible system models to ours. The system we study is quite comprehensive in that there are multiple users that are mobile, and multiple ESs. The tasks are generated not simultaneously, or periodically, but continuously in a stochastic manner. Many works in the literature consider only a snapshot of a system, where a number of users all have a single task each, and optimization problems are formulated based on these snapshots. Although these are valuable works, they do not speak to the systems where tasks arrive at random time instants in a stochastic manner since such systems would be very hard to investigate using static optimization tools.

It proved to be quite a challenge to identify a study that could be suited as a benchmark as many studies either lack a crucial component our model has or involve components that we do not consider, such as additional cloud servers or physical-layer components such as beamforming, which render them incompatible for a comparison, as summarized in Table 1. The model described in [7] seems to be the only compatible system in this list, except for user mobility. It features multiple servers and users, each user generating multiple tasks, and does not involve any additional cloud servers. This work (which we call *the benchmark* in the sequel) was used as a comparison benchmark, and we describe the main differences between the two models and how we made them compatible in this section. (Note that the modifications described here only apply to the part of the simulation study where a comparison between our results and the benchmark is made and not to the rest of the simulation study).

#### 3.6.1. Task Characteristics

In the benchmark, the servers are not modeled as processing units with frequency values. Rather, an M/M/c queueing model is employed, and thus tasks are characterized as jobs or clients arriving to an M/M/c queue. Instead of the usual data size and required CPU cycle characterization observed in the literature (including our work), the benchmark describes the tasks with their size, which are assumed constant, and their service time requirements, which have exponential distributions. To be compatible with the benchmark, we matched the aggregate load on both systems. To achieve this, we first pick a suitable $f_{s}$, set $n_{c}=1$ and p=1, fix *D* to the data size used in the benchmark, and produce *C* values for each task using an exponential distribution with an appropriate mean value to match the mean service time used by the benchmark.

#### 3.6.2. Mobility

The users are stationary in the benchmark. Therefore, we also used stationary UE in the simulation study for the comparison.

#### 3.6.3. Communication Model of the Benchmark

In the benchmark, the received power is expressed as

(11) $$P_{R}=P_{T}\;{\left|h\right|}^{2}\;d^{-\alpha },$$

where *h* is the channel coefficient, *d* is the distance between the transmitter and the receiver, and α is the path loss exponent, which is assumed to be different for uplink communications and on the wireless backhaul. The distance used in the benchmark is two-dimensional, and thus we ignored antenna heights for the comparison. *h* is taken as 1 in the benchmark, so it does not have a numerical effect on the results. Furthermore, the benchmark does not take into account the antenna gain and shadow fading. Therefore, we use (11) instead of (1) and (2) for the comparison. Finally, the benchmark assumes that the bandwidth allocated to each BS is shared between the users connected to that particular BS, proportional to their task generation rate that is assumed to be known.

#### 3.6.4. Computation and Queueing Models

In the benchmark, a certain number of ESs are distributed to a number of BSs to be deployed, and the resulting system is modeled as M/M/c queues operating according to the FCFS discipline at each of these BSs. An optimization problem is formulated for the distribution of the ESs into the BSs in the benchmark, and we also used the solution presented in the benchmark in terms of the placement and locations of the ESs.

One significant aspect of the comparison is the difference that the queueing discipline makes. Typically, PS performs better in terms of the sojourn time than FCFS, provided that the coefficient of variation of the service time (or job size) distribution exceeds 1 [45] (p. 483). This condition is mostly satisfied in the simulation scenarios, and thus the PS discipline provides an advantage in the numerical results.

#### 3.6.5. Offloading and Edge Server Selection Models

The benchmark formulates an optimization problem to minimize the average delay and produces both the server placement (i.e., how many servers to deploy at each BS) and the offloading association (i.e., which user offloads to which BS) as the result. Therefore, the ES selection is completed on a per-user basis rather than a per-task basis as in our study. Furthermore, users are assumed to always offload and never compute locally in the benchmark. To ensure this, the computing capacity of the UE was selected to be sufficiently low so that all the tasks would be offloaded. This scenario could represent a system where the users are not expected to complete any computation, such as a sensor network scenario.

## 4. Numerical Experimentation

**Figure 2:**
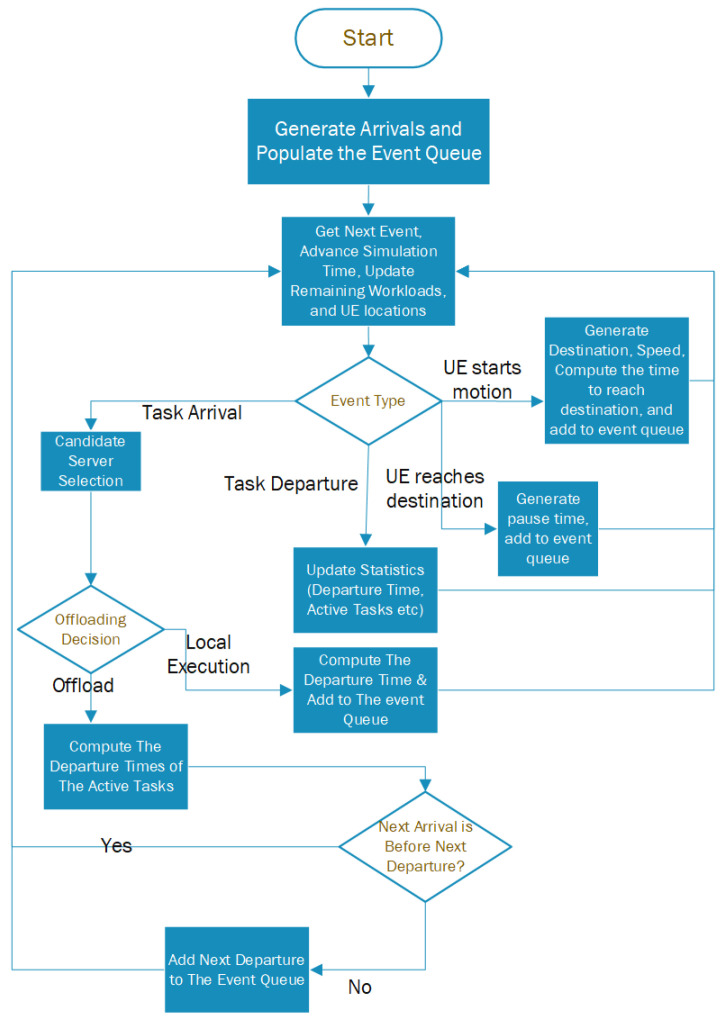
Simulation flowchart.

For the purpose of this study, a standalone simulator was written in Matlab. Figure 2 illustrates the simulation process, which begins by generating task arrivals following a Poisson process with the specified arrival rate. The event queue is populated with these arrivals. The simulation then proceeds by determining the timing of the next event, advancing the simulation clock, and updating the system workload. Each event may represent a task arrival, a task departure, a UE initiating movement, or a UE reaching its destination. In the case of UE starting motion, the destination and speed are generated, and then the time to reach the destination is computed and added to the event queue. If the event is UE reaching the destination, a pausing time is generated and added to the event queue. In the case of a departure, relevant statistics such as the departure time, the number of active tasks in the system, and the total count of processed tasks are updated. For arrival events, offloading decisions are made based on the estimated sojourn time. If local execution is determined to provide superior service, the task is scheduled for local processing, its departure time is computed, and the event is added to the event queue. Conversely, if offloading is deemed more advantageous, the ES is selected using the designated server selection scheme. Following this, the departure times for all active tasks are calculated and the next departure event is added to the event queue. The simulation then advances to the next event, and the process is repeated iteratively.

The simulation studies conducted can be separated into two main sets. In the first set of simulations, we compare the performance of the ES selection methods described earlier to the results of the benchmark [7] under the simulation settings of the benchmark. In terms of the geographical layout of the simulation area and the placement of the BSs, we used a real-world dataset obtained from the panOULU public network, spanning the years 2004 to 2013, in the city of Oulu, Finland [46], as conducted in the benchmark. To simplify the simulations, the network size is proportionally reduced, with the maximum distance between the two farthest users scaled down to 1.5 km. Additionally, the original 1400 panOULU BSs are reduced to 36 BSs, whose locations are determined using the 36-means clustering algorithm. The panOULU BSs are grouped into 36 clusters, and the centroids of these clusters serve as the BS locations in the scaled down network. Users are randomly distributed according to the population data for Oulu. This entire process of building the simulation area directly follows the benchmark [7], and both sets of simulations (including the second set that does not involve the benchmark) use this geography.

### 4.1. Comparison with the Benchmark

In this section, we report the numerical results for the comparison of the performance of the server selection methods described in Section 3.5 to the benchmark [7]. The simulation setup is built as described in Section 3.6. Unless otherwise specified, the simulation parameters follow those listed in Table 2, consistent with [7]. In particular, the benchmark assumes a service rate of 2.5 tasks per second at the M/M/c queues. To match this, task sizes are taken as 9.5 MB as in the benchmark, the CPU frequency of each server is assumed to be 2 GHz, and the number of required CPU cycles per bit is assumed to have an exponential distribution with mean 100/9.5. This results in exponentially distributed service times with $\frac{2\times 10^{9}}{\left(8\times 9.5\times 10^{6}\right)\times \left(100/9.5\right)}=2.5$ tasks completed per second on average, compatible with the benchmark.

**Figure 3:**
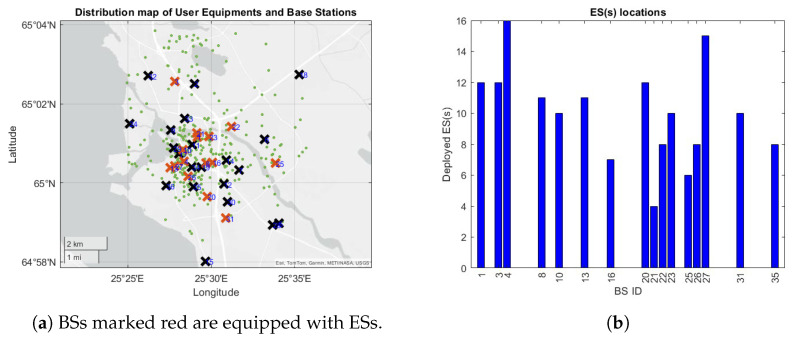
(**a**) BS (crosses) and UE (green dots) positions according to the benchmark, and (**b**) the number of deployed ESs at each BS.

Figure 3a shows the distribution of BSs, the placement of ESs, and the user locations, as determined in the benchmark. BSs with deployed ESs are marked in red, and their BS identification numbers are shown. The BSs marked black do not have any ESs installed. Green dots represent UE locations. The number of ESs deployed at each selected BS is indicated in Figure 3b.

**Figure 4:**
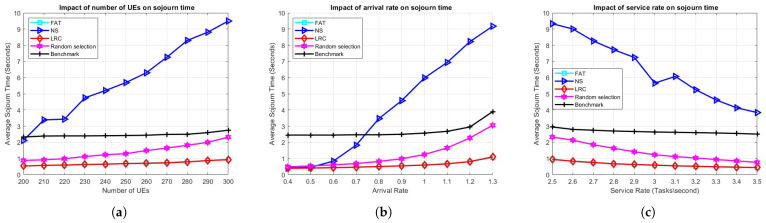
The effect of (**a**) the number of UE, (**b**) the arrival rate, and (**c**) the service rate on the average task sojourn time in the benchmark scenario.

Figure 4 presents a comparative analysis of the ES selection methods described in Section 3.5 as well as the benchmark. Figure 4a demonstrates the effect of user population on the average task sojourn time. Evidently, both LRC and FAT exhibit superior performance compared to the other schemes, including the benchmark. This result can be attributed to the benchmark’s use of the FCFS queueing discipline, whereas our approach employs round-robin scheduling. Notably, the random selection scheme performs better than the benchmark, which underscores the effectiveness of round-robin scheduling in optimizing overall system performance. Another factor in the benchmark being outperformed is that the ES that will execute the tasks generated by each UE is fixed in the benchmark, regardless of the instantaneous load on the selected ES as well as others that may have lighter loads. This results in potentially unused capacity in the overall system. LRC and FAT have comparable performances since the task characteristics are somewhat static, and the ESs with the lightest loads in terms of the number of remaining cycles and the number of active tasks are typically the same. Conversely, NS exhibits the poorest performance among all schemes. This is likely due to the asymmetric network topology, the considerable distance between certain BSs and the deployed servers, and the nonuniform distribution of users. These factors contribute to an imbalance in load distribution, where some ESs become overloaded while others remain idle, ultimately leading to increased delays for users.

Figure 4b illustrates the impact of arrival rate on the average sojourn time. It can be seen that LRC, FAT, RS, and NS exhibit comparable performance under low-load conditions, with delays that remain below one second. However, as the load increases, particularly when the arrival rate reaches 0.6 tasks/s, the performance of NS and RS begins to degrade, significantly in the case of NS. Among these, NS demonstrates the poorest performance, with delays escalating to approximately 9 s at higher loads. In contrast, LRC and FAT maintain superior and nearly identical performance, achieving delays of no more than 1 s even at a load of 1.2 tasks/s. This highlights the robustness and efficiency of LRC and FAT under varying load conditions.

Figure 4c illustrates the effect of the service rate on the sojourn time. Once again, LRC and FAT exhibit nearly identical performance. RS and the benchmark start with sojourn times of approximately 2.3 and 3 s, respectively, while NS begins at around 9.3 s. As the service rate increases, the delay experienced by users decreases in all the methods. NS demonstrates a significant response to the increased service rate, with its sojourn time decreasing to approximately 4 s. In contrast, the benchmark, LRC, and FAT show minimal sensitivity to the increased service rate. This is because the initial service rate of 2.5 tasks/s is already sufficient to handle the offloaded tasks without causing serious server overloads.

The results presented in this section demonstrate that the proposed offloading strategy along with LRC or FAT as the ES selection scheme perform very well in comparison to the benchmark. It should also be noted that the algorithm presented in the benchmark was reported to outperform five more algorithms in the given scenario [7], so the proposed offloading strategy can be considered to outperform not a single but at least six methods existing in the literature.

### 4.2. Comparison of the ES Selection Methods

After we have shown that the proposed offloading scheme performs significantly better than the benchmark using certain ES selection methods, we turn our attention to the comparison of these ES selection methods. In this second part of the simulation study, the users are evenly divided into two classes to account for the variety in user applications: Class 1 generates lightweight tasks, while Class 2 generates computationally intensive tasks. In contrast to the benchmark study, each user is now allocated a bandwidth of 100 MHz for transmitting data through the communication channel to and from the BS, while a bandwidth of 500 MHz is available in the backhaul. Furthermore, except for the first scenario forthcoming, users move within the simulation area following the RWP mobility model, with speeds ranging from 0 to 50 m per second. Each server is equipped with a CPU containing 24 cores, as described in [47]. Unless otherwise stated, the remaining simulation parameters are used as listed in Table 3.

Before delving into more general scenarios with user mobility under our system model, we compared the ES selection schemes with stationary users. In this scenario, 64 ESs are deployed, with 4 ESs allocated at each selected BS. The selected set of BSs are the same as indicated in Figure 3a.

**Figure 5:**
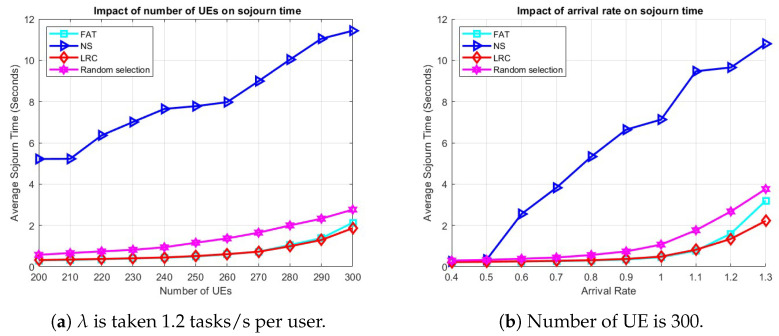
The effect of (**a**) the number of UE and (**b**) arrival rate on sojourn time in a scenario with 64 ESs and stationary UE.

Figure 5a demonstrates the influence of the number of UE on the average sojourn time. In particular, LRC and FAT maintain very similar performance levels, even as the number of users increases, which inherently raises the load on the servers. Both LRC and FAT initially achieve a sojourn time of approximately 0.3 s when the number of users is 200. However, LRC exhibits a slight performance edge as the user count reaches 300, underscoring its superiority under high-load conditions compared to all other methods. On the other hand, NS performs much worse than even RS, which starts at around 0.7 s with 200 users and gradually increases to approximately 3 s as the user count reaches 300. NS, once again, emerges as the least-effective approach, with a notable performance disparity relative to the others. It begins at approximately 5.3 s and progressively rises as the number of users grows, reaching around 11.7 s at 300 users. This underscores the inefficiency of NS in handling increased user loads, primarily due to highly unbalanced server loads, which leads to higher computational delays experienced by users.

Figure 5b shows the impact of task arrival rate on the average sojourn time. At low arrival rates, all methods exhibit comparable performance. However, as the arrival rate increases, particularly beyond 0.5 tasks per second, the performance of NS begins to degrade significantly, exceeding 10 s when the load reaches 1.3 tasks per second. When the load approaches 0.8 tasks per second, RS also starts to show a decline in performance, while LRC and FAT maintain steady and consistent results. When the arrival rate surpasses 1.2 tasks per second, LRC demonstrates clear dominance over the other approaches, showcasing its superior ability to manage and balance high loads across servers. This underscores LRC’s effectiveness in enhancing system performance compared to alternative server selection methods.

**Figure 6:**
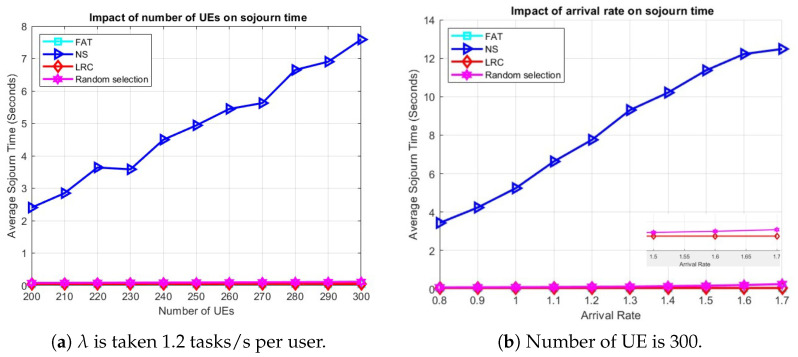
The effect of (**a**) the number of UE and (**b**) the arrival rate on sojourn time, respectively, in a scenario with user mobility.

To see how things play out in case of user mobility, the average sojourn times for varying numbers of UE and arrival rates are given in Figure 6. The simulation parameters in Table 3 were used, with one ES deployed at each of the 36 BSs and users roaming the network according to the RWP mobility model. The user base was divided into two classes, where 50% of the total users generate light tasks and the remaining 50% generate heavy tasks, with task sizes as specified in Table 3 and uniform intensities $\left[10^{3},3\times 10^{3}\right]$ CPU cycles per bit for light tasks and $\left[4\times 10^{3},7\times 10^{3}\right]$ CPU cycles per bit for heavy tasks.

Figure 6a demonstrates the influence of the number of UE on the average sojourn time with an arrival rate of 1.2 tasks/s per user. Among all the methods, NS exhibits the poorest performance, primarily due to the asymmetric distribution of BSs, which results in certain BSs becoming overloaded. For instance, as illustrated in Figure 3a, BSs such as BS 15, BS 18, and BS 24 lack neighboring BSs, compelling all the UE in those areas to offload tasks to these BSs and causing the associated ESs to become overloaded. Figure 6b illustrates the effect of arrival rate on the average sojourn time when the number of users is 300. Consistent with previous findings, NS continues to underperform for the reasons previously stated. The performance of RS also begins to deteriorate slightly as the arrival rate increases. LRC and FAT remain effective under higher-load conditions. RS performing not so far off from the LRC and FAT duo can be attributed to it implicitly balancing the load by randomly assigning tasks to different ESs.

**Figure 7:**
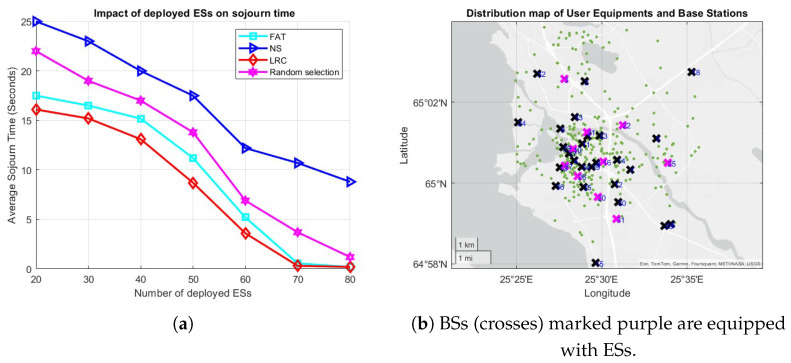
(**a**) The effect of the number of ESs on the average sojourn time, and (**b**) the distribution of ESs, BSs, and the UEs (green dots).

To evaluate the impact of the number and distribution of the ESs deployed in the network on the performance of the proposed approaches, 10 randomly selected BSs were equipped with equal number of ESs, as illustrated in Figure 7b. These results were observed under an arrival rate of 1.3 tasks/s per user. Notably, as shown in Figure 7a, when 2 ESs are deployed per selected BS (20 in total), LRC outperforms all the other methods, achieving an average sojourn time of approximately 16 s, while FAT performs slightly worse, with an average sojourn time of around 17.5 s. As the number of deployed ESs increases, the sojourn time decreases across all the methods. Specifically, when 70 to 80 ESs are deployed, both LRC and FAT demonstrate superior performance, with average sojourn times of approximately 0.2 s, while NS once again performs the worst among all the methods. This observed behavior of NS can be attributed to inefficient load balancing across the servers, as well as the increasing distances between mobile users and their associated ESs, which lead to significant data transmission delays in both the backhaul and communication channels.

**Figure 8:**
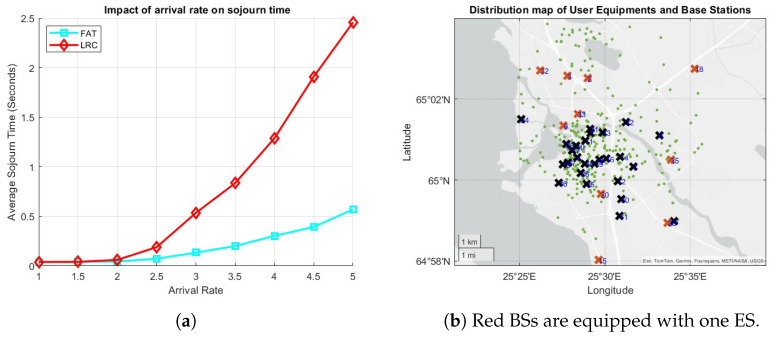
(**a**) The effect of the arrival rate on the average sojourn time, and (**b**) the distribution of ESs, BSs (crosses), and the UEs (green dots).

Although LRC has shown the best performance in the scenarios above, we show that it is not the absolute winner with the next scenario. For this final experiment, 10 BSs are randomly selected, each of which is equipped with one ES, denoted by red crosses in Figure 8b. In this scenario, 99% of the users generate light computational tasks with CPU cycle requirements ranging between $\left[10^{3},3\times 10^{3}\right]$ cycles per bit, while the remaining 1% generate heavy tasks requiring $\left[10^{5},3\times 10^{5}\right]$ cycles per bit, meaning that there is a 100-fold workload difference between light and heavy tasks on average, while light tasks are generated 99 times more frequently. The average task sojourn times under this scenario for FAT and LRC are presented in Figure 8a. At low task arrival rates, both FAT and LRC achieve similar delays, maintaining an average sojourn time of around 0.1 s. As the arrival rate increases beyond 2 tasks per second, FAT demonstrates superior performance. Specifically, at an arrival rate of 5 tasks per second, FAT sustains a significantly lower average delay (approximately 0.6 s), whereas LRC experiences a substantially higher delay (around 2.5 s). This difference can be attributed to the task allocation strategies employed by each approach. Under LRC, a server processing a heavy task is effectively excluded from receiving additional tasks as the remaining CPU cycles required for completion are, on average, 100 times greater than those of a light task. Consequently, incoming tasks are directed to other servers that may already be handling multiple light tasks. Furthermore, if another heavy task arrives, it is assigned to a different server, further exacerbating the accumulation of tasks in the remaining servers. Under round-robin scheduling, the computational resources of the server are equally distributed among the piled-up light tasks, leading to increased delays, particularly under high arrival rates. In contrast, FAT employs a server selection mechanism based on the number of active tasks per server. As a result, a server processing a heavy task remains eligible to receive additional incoming tasks. Under this mechanism, computational capacity is equally allocated among all the active tasks, both light and heavy. Since light tasks require significantly fewer cycles, they are processed and depart the system much more quickly, preventing the accumulation observed in LRC. This dynamic allows FAT to maintain lower delays compared to LRC, particularly as task arrival rates escalate.

## 5. Discussion and Conclusions

In this study, we present an offloading framework for mobile edge computing systems with multiple ESs that employ round-robin task scheduling. We also conduct a comparative analysis of a number of server selection schemes, including fewest active tasks (FAT), in which an offloaded task is assigned to the ES with the fewest number of tasks actively running on it, and least remaining CPU cycles (LRC), in which an offloaded task is assigned to the ES with the least amount of workload in terms of the number of CPU cycles remaining, against three other approaches, namely the nearest server, random selection, and a compatible study from the literature. The numerical experiments reveal the following conclusions:

- FAT and LRC consistently outperform all the other methods. This is not surprising as both methods are variants of the join-the-shortest-queue approach, known to perform well in multiserver scenarios. Moreover, we quantify the magnitude of the performance gain.
- The performance of FAT and LRC is not significantly different as long as there is not large variation in the task sizes.
- Given that the simulations were conducted in a realistic asymmetrical network setting, the results can be interpreted as representing a worst-case scenario for the NS scheme. NS could achieve more balanced loads and improved performance in a symmetrical topology. However, in an asymmetrical scenario, even random selection outperforms the NS method.
- In terms of task delay, round-robin scheduling performs much better compared to FCFS, which is preferred in analytical studies due to its simplicity and closed-form solutions. However, relying on FCFS formulations in order to formulate optimization problems can be misleading.

Further research will explore the integration of round-robin-based task scheduling into more complex scenarios, possibly involving resource allocation schemes with physical-layer considerations.

## Figures and Tables

**Table 2:** Simulation parameters for the benchmark study.

Parameters	Values
Number of UE	300
Number of BSs	36
Number of BSs hosting ESs	16
Total number of ESs	160
Service rate of each server	2.5 tasks/s
Task arrival rate	1.2 tasks/s
Offload bandwidth	100 MHz
Backhaul link bandwidth	500 MHz
Noise spectral density	−125 dBm/Hz
Task size	9.5 MB
Result size	0.5 MB
Required CPU cycles per bit	Exp(100/9.5)
User transmit power	200 mW
BS transmit power	1 W
Path-loss exponent (offload)	3
Path-loss exponent (migrate)	2.5
$f_{s}$	2 GHz
$f_{m}$	1 KHz

**Table 3:** Simulation parameters for the ES selection method comparison.

Parameters	Values
Number of UEs	300
Number of BSs	36
UE speed	Uniform[0,50] m/s
RWP pause time	Uniform[0,10] s
Task data size, Class 1	Uniform[100,300] KB
Required CPU cycles per bit, Class 1	Uniform$\left[10^{3},\;5\times 10^{3}\right]$
Task data size, Class 2	Uniform[400,600] KB
Required CPU cycles per bit, Class 2	Uniform$\left[10^{4},\;2\times 10^{4}\right]$
Result size	0.5 MB
λ (tasks/s per user)	1.2
$f_{s}$	3.5 GHz
$f_{m}$	2 GHz
$n_{c}$	24
*p*	2
θ	0.2
$P_{N}$	−106 dBm
$P_{T}$	19.64 dBm
*G*	20 dB
$f_{c}$	30 GHz
$h_{\mathrm{BS}}$	25 m
$h_{\mathrm{UT}}$	1.5 m
$\sigma_{SF}$	7.8 dB
Offload bandwidth	100 MHz
Backhaul link bandwidth	500 MHz

## Data Availability

The original data used in the study are openly available from Oulu City Data Portal at https://data.ouka.fi/data/en_GB/dataset/panoulu (accessed on 1 April 2025).

## Abbreviations

The following abbreviations are used in this manuscript:

BS	Base station
ES	Edge server
FAT	Fewest active tasks
FCFS	First-come-first-served
LRC	Least remaining CPU cycles
MEC	Mobile edge computing
NS	Nearest server
PS	Processor sharing
RS	Random selection
RWP	Random waypoint
SNR	Signal-to-noise ratio
SP	Service provider
UE	User equipment
